# Addressing ethical issues in H3Africa research – the views of research ethics committee members

**DOI:** 10.1186/s11568-015-0006-6

**Published:** 2015-01-31

**Authors:** Jantina de Vries, Akin Abayomi, Katherine Littler, Ebony Madden, Sheryl McCurdy, Odile Ouwe Missi Oukem-Boyer, Janet Seeley, Ciara Staunton, Godfrey Tangwa, Paulina Tindana, Jennifer Troyer, The H3Africa Working Group on Ethics

**Affiliations:** Department of Medicine, University of Cape Town, Old Main Building, Groote Schuur Hospital, Observatory, 7925, Cape Town, South Africa; NSB-H3A biobank, National Health Laboratory Services of South Africa, Faculty of Medicine and Health Sciences, Stellenbosch University, Tygerberg Hospital, Cape Town, 7505 South Africa; Wellcome Trust, Gibbs Building, 215 Euston Road, London, NW1 2BE UK; Division of Genomic Medicine, National Human Genome Research Institute, National Institutes of Health, Building KEYSTN, Room 3130, 530 Davis Dr, MSC K3-02, Durham, NC 27713-K3-02 UK; Center for Health Promotion and Prevention Medicine, School of Public Health, University of Texas, 7000 Fannin, Suite 2056D, Houston, TX 77030 USA; CERMES, 634 Bd de la Nation, PO Box 10887, YN034 Niamey, Niger; Cameroon Bioethics Initiative (CAMBIN), the Ark, Mendong, PO Box 31489, Yaoundé, Cameroon; MRC/UVRI Uganda Research Unit on AIDS, P.O. Box 49, Entebbe, Uganda; Centre for Medical Ethics and Law, Department of Medicine, Faculty of Medicine and Health Sciences, Stellenbosch University, Francie van Zijl Drive, TYGERBERG, PO Box 19063, Stellenbosch, 7505 South Africa; Department of Philosophy, University of Yaounde 1, P.O. Box 13597, Yaounde, Cameroon; Navrongo Health Research Centre, Ghana Health Service, P.O.Box 114, Navrongo, Ghana; Human Heredity and Health in Africa, National Human Genome Research Institute, NIH, 5635 Fishers Lane Suite 4076, Rockville, Maryland 20852 USA

**Keywords:** H3Africa, Ethics, Genomics, Africa, Broad consent, Community engagement, Sample sharing, Biobanking

## Abstract

In June 2014, the H3Africa Working Group on Ethics organised a workshop with members of over 40 research ethics committees from across Africa to discuss the ethical challenges raised in H3Africa research, and to receive input on the proposed H3Africa governance framework. Prominent amongst a myriad of ethical issues raised by meeting participants were concerns over consent for future use of samples and data, the role of community engagement in large international collaborative projects, and particular features of the governance of sample sharing. This report describes these concerns in detail and will be informative to researchers wishing to conduct genomic research on diseases pertinent to the African research context.

## Introduction

Genomic research in Africa raises a number of unique ethical challenges, arising most prominently from the interplay between vanguard science and traditional communities and research contexts. H3Africa research, which seeks to foster genomic research on diseases pertinent to African people, needs to carefully consider these ethical challenges. In June 2014, the H3Africa Working Group on Ethics convened a consultative meeting with members of research ethics committees (RECs) from across Africa to discuss ethical challenges in H3Africa research, with particular focus on issues relating to broad consent, sample and data sharing. H3Africa is an international collaboration of scientists working to build genomic research capacity in Africa (H3Africa Consortium 2014). The goal is to support cutting edge research to advance understanding of the genetic and environmental determinants of common diseases in Africa and to use this knowledge to improve the health of African populations. A key component of H3Africa research is the global sharing of data and selected biospecimens to promote their utility and to speed up discovery of new knowledge that could impact disease prevention and management. This raises a number of key ethical considerations that need to be addressed in order for H3Africa research to be successful. To better understand these issues and the perspectives of research ethics committees on H3Africa research, the H3Africa Working Group on Ethics hosted a consultation meeting to which we invited the Chairs of research ethics committees involved in the review of H3Africa projects. The meeting was attended by just over 80 people including 60 members of 40 research ethics committees from 18 African countries.^1^ Also in attendance were a number of H3Africa researchers, members of the H3Africa Working Group on Ethics, and representatives of the two funding agencies that are supporting H3Africa research; the US National Institutes of Health and the Wellcome Trust. In this report, we share the main lessons learnt from this consultation meeting.

### Broad consent

Much of the days’ discussion focussed on consent in general and consent for genomic research specifically. Meeting participants referred to traditional knowledge about inheritance – for instance, that particular disease traits are more common in some families – as a way to explain genomic research. The most pertinent topic of discussion, however, related to consent for sample sharing and biobanking. The proposal is that H3Africa samples will be deposited in one of four H3Africa Biorepositories – based on the African continent – for onward distribution to the scientific community. Similarly, and in line with international standards, genomic data will be deposited in data repositories for secondary use. From a scientific and funder perspective, it is recommended that H3Africa samples and data are collected with broad consent to allow for the sharing of genomic and phenotype data as well as human samples (see for instance the H3Africa Guidelines on Informed Consent, www.h3africa.org). In the H3Africa Guidelines on Informed Consent, broad consent means consent that allows use of samples and genomic and phenotype data for future research with ethics approval and the possibility to withdraw. In the scientific genomic community, this sort of consent is commonly referred to as ‘broad consent’. As the presentations and discussions ensued it became apparent that this definition was not shared by the workshop participants who alternately spoke of ‘open’ , ‘blanket’ and ‘unrestricted’ consent. In the understanding of the H3Africa Working Group on Ethics, these latter terms refer to consent that does not place any limitations on the kinds of research that samples and data can be used for in future and whereby secondary use takes place without ethics approval. Consent without limitations was considered to possibly be unethical by many of the meeting participants because it would not constitute ‘informed’ consent. The term broad consent in the bioethics community indicates consent to a wide range of research topics, including for instance to biomedical, genomic, and population genetics research projects, that have ethics approval. Broad consent does not imply that samples and data can be used for absolutely anything. Meeting participants felt that consent that allows for some future use could be permissible in the African research context, providing that it is valid and clearly understood. By ‘African research context’ we mean a highly diverse research setting that is characterised by often under-resourced healthcare and research systems servicing a mixed urban and rural population, of varying degrees of wealth, with varying degrees of literacy and with a high burden of communicable and non-communicable diseases and with diverse national laws. There was concern that where valid consent is already difficult to obtain due to poverty and low education, as is the case in many African research contexts, seeking ‘broad’ consent in addition to specific consent for the primary research project poses real challenges. Some meeting participants also suggested that research participants should have an option to refuse consent for future use whilst being allowed to participate in the primary research project. If participants do not have an option to refuse sample sharing and future re-use then some of the meeting participants felt that this could compromise the validity of consent, particularly because people often give consent on the basis of trust, not knowledge.

It was clear during the meeting that there is vast diversity in experience between the committee members and countries represented. For instance, the Ugandan National Council for Science and Technology has been using and approving broad consent for the past six years, whereas members of the other committees indicated that they had not reviewed any projects using broad consent. Also, it became clear that within particular countries there may be legal limits to the permissibility of broad consent, and to the timeframe for storage of samples. Following from these discussions, it is clear that the consent requirements for projects like H3Africa that involve the sharing of samples and data for secondary use need to be better understood in general, and specifically in the African research context. There is an urgent need for empirical work that explores the views of a variety of stakeholders to investigate what they understand broad consent to be. There is also a need to outline the criteria under which broad consent can (not) be used in medical research in Africa. Such empirical work is needed to inform both practice and policy development in this area.

### Community engagement

Quite prominent in the discussions was the role of community engagement to support both broad consent and the governance of biobanks. Community engagement is very important in the African research context, particularly when research participants live in rural communities that are isolated from information about genomic research and health care facilities, and that are embedded in long-standing support systems and values. In rural communities especially, the values and concerns of the community are often considered to be at least as important as individual values prioritized in the informed consent documents. Community engagement is one way of considering community values in research, and is an important step in respecting communities. The meeting participants indicated that involvement of communities throughout the research process – preceding and during sample collection and in future use decisions – is of key importance in ensuring ethical conduct in research. The strong emphasis on community engagement, and the proposal that communities are involved in biobank governance in some way, is a unique feature of the African research context. As a minimum, it was suggested that the governance framework for sample and data sharing ought to be informed by community engagement. But community engagement raises many questions, for instance about what constitutes a ‘community’ and who represents it, how it can effectively be consulted and involved, and how community engagement can be evaluated. There is also a need to better understand the applicability of community engagement long-term, for instance in biobank governance. A particular challenge in the H3Africa context is that H3Africa biobanks will be organised internationally, and that samples from a multitude of communities will be stored in them. Exactly how communities can and should be involved in the governance of the H3Africa biobanks is an important question that will need to be investigated.

### Governance framework for biobanking research

During the meeting, the need for a good governance framework to guide sample and data access decisions became exceedingly clear. The purpose of a transparent and robust governance framework would be to build trust, prevent harm and maximise benefit. The framework needs to address essential questions about control over and ownership of samples and data. It would also need to specify whether and to which extent commercialisation of samples or sample products are permissible. The framework would need to comply with local and national guidelines and regulations insofar as these exist. Guidance would have to articulate a clear mechanism for withdrawal of samples and data from the biorepository and ensure that confidentiality is maintained throughout the research process. Of note is the virtual absence of guidance on biobanking research in African jurisdictions. Many of the research ethics committee members present at the meeting felt that they needed such guidance to help them review project proposals involving sample and data sharing. Such guidance should specifically take into consideration the African research context, which includes infrastructural challenges, instability in governments, Africa’s colonial history and continued use of the expertise of local medical healers who work with plants and the spirit world within a system that attaches symbolic value to blood and other human samples.

It was clear from the discussions that there is a need to continue to involve the research ethics committees that originally approved sample collection in sample sharing decisions in some way. As a minimum, this could be through regular reporting of the biobanking activities. It was also proposed that research ethics committees could indicate when particularly vulnerable population groups are involved in research and request to be party to sample access decisions in those cases. Also, it should be possible for the people deciding on sample access to refer difficult secondary access proposals to the original research ethics committee that approved sample collection. It became clear during the meeting that there is a continuing need for training of research ethics committee members in the particulars of genomic and biobanking research.

The H3Africa Consortium has just finalised the Terms of Reference of the H3Africa Data and Biospecimen Access Committee (for more information see www.h3africa.org). These terms were still under development when the Ethics Consultation Meeting was held and they were amended as a direct outcome of this meeting. Two important changes were introduced: first, the terms now stipulate mandatory regular reporting of access decisions to the research ethics committees that approved sample collection, and second, they introduce the possibility for the access committee to consult with members of the research ethics committee that originally approved sample collection if and when necessary.

### Conclusion and ways forward

There are multiple challenges confronting genomic research in Africa and these have been highlighted in the H3Africa Marker Paper (H3Africa Consortium 2014). Pertinent amongst these challenges are ethical issues. The H3Africa Ethics Consultation Meeting was a critical step in understanding better the range of issues related to H3Africa research, including the question “How will Africa benefit? a point of critical importance to both presenters and meeting participants. By engaging members from research ethics committees and national ethics councils from 18 countries we began a much needed dialogue about ways to coevolve science and ethical regulation in the African research context. In this report, we have highlighted the most pertinent issues that were discussed during the H3Africa Ethics Consultation Meeting, namely challenges relating to consent, community engagement and the governance of sample and data access. Most clearly, the meeting established the centrality of trust in ensuring that H3Africa research comes to fruition. In order for the sharing of samples and data to be ethically acceptable in the African research context, the research needs to be accompanied by thorough community engagement and embedded in a robust and transparent governance framework. Samples and data also need to be collected with valid consent. In addition to these ethical issues, challenges were identified that are not new to H3Africa research but that do affect it. These were for instance issues relating to inducement, monitoring of approved research and how to explain complex scientific concepts during the consent process.

H3Africa has established a Working Group on Ethics that seeks to systematically interrogate these issues and help develop an ethical framework for genomic research in Africa. As a Working Group the meeting helped us realise the value of ongoing engagement with those involved in research ethics review and hope to continue this activity. Specifically, this meeting helped the Working Group to develop a strategic workplan on informed consent. As part of this workplan, we are analysing current H3Africa consent forms used by the various projects to examine the range of consent models used. We have also sourced ethics guidelines and legislation from a variety of African countries to examine ethical and legal perspectives and obstacles to genomics and biobanking research. The Working Group also managed to secure funding for a follow-up meeting with REC members to specifically explore the ethical challenges around informed consent for biobanking and genomics research. This meeting is likely to be held in Zambia in May 2015. Several empirical projects on informed consent are also being conducted in several African countries.

With regards to the training of REC members in the review of genomics and biobanking research, the H3Africa Working Group Ethics has teamed up with the West African Bioethics Network to develop a hybrid online and in-person training course. Funding for curriculum development has been received from the NIH and course materials are currently being developed and should become available online in August 2015.

With regards to Community Engagement, the Working Group has just finalised a project that sought to identify different community engagement models that can be used to support African genomics research. The first phase of the project which involved a literature review was submitted for publication and will hopefully be published in 2015. The second phase involved an assessment of community engagement activities taking place within H3Africa research projects which consisted of interviews and a questionnaire to H3Africa researchers. The results of this project have been written up into a research report that is being finalised by the Working Group. More information about the work of this group, a more complete version of these meeting proceedings and a list of meeting participants can be found at www.h3africa.org.

## Endnote

^a^Participants came from Benin, Botswana, Burkina Faso, Cameroon, Cote d'Ivoire, Ethiopia, Ghana, Guinea, Kenya, Malawi, Mali, Mozambique, Nigeria, South Africa, Sudan, Tanzania, Uganda, Zambia.

## References

[CR1] H3Africa Consortium (2014). Research capacity. Enabling the genomic revolution in Africa. Science.

